# Evaluation of the Geriatric Trauma Outcome Score (GTOS) as a Prognostic Tool in Intensive Care Unit Trauma Patients

**DOI:** 10.3390/diagnostics14192146

**Published:** 2024-09-26

**Authors:** Ching-Ya Huang, Shao-Chun Wu, Hang-Tsung Liu, Wei-Ti Su, Shiun-Yuan Hsu, Chi Li, Ching-Hua Hsieh

**Affiliations:** 1 Department of Plastic Surgery, Kaohsiung Chang Gung Memorial Hospital and Chang Gung University College of Medicine, Kaohsiung 83301, Taiwan; b101106030@tmu.edu.tw; 2 Department of Anesthesiology, Kaohsiung Chang Gung Memorial Hospital and Chang Gung University College of Medicine, Kaohsiung 83301, Taiwan; shaochunwu@gmail.com; 3 Department of Trauma Surgery, Kaohsiung Chang Gung Memorial Hospital and Chang Gung University College of Medicine, Kaohsiung 83301, Taiwan; htl1688@yahoo.com.tw (H.-T.L.); s101132@adm.cgmh.org.tw (W.-T.S.); ah.lucy@hotmail.com (S.-Y.H.)

**Keywords:** trauma, intensive care unit (ICU), geriatric trauma outcome score (GTOS), prognosis, mortality

## Abstract

Background: Existing prognostic scoring systems for intensive care unit (ICU) trauma patients require extensive data collection. The Geriatric Trauma Outcome Score (GTOS), which is based on age, injury severity, and transfusion need, has been validated for predicting mortality in elderly patients with trauma; however, its utility in the general ICU trauma population remains unexplored. Methods: This retrospective study included 2952 adult ICU trauma patients admitted between 2016 and 2021. The GTOS was calculated as follows: age + (Injury Severity Score × 2.5) + 22 (if transfused within 24 h). The area under the receiver operating characteristic curve (AUROC) was used to assess GTOS’s ability to predict mortality. The optimal GTOS cutoff was determined using Youden’s index. Mortality rates were compared between the high and low GTOS groups, including a propensity score-matched analysis adjusted for baseline characteristics. Results: This study included 2952 ICU trauma patients, with an overall mortality rate of 11.0% (*n* = 325). GTOS demonstrated good predictive accuracy for mortality (AUROC 0.80). The optimal cutoff was 121.8 (sensitivity, 0.791; specificity, 0.685). Despite adjustments, patients with GTOS ≥ 121.8 had significantly higher mortality (17.4% vs. 6.2%, *p* < 0.001) and longer hospital stays (20.3 vs. 15.3 days, *p* < 0.001) compared to GTOS < 121.8. Conclusions: GTOS showed a reasonable ability to predict mortality in ICU trauma patients across all ages, although not as accurately as more complex ICU-specific models. With its simplicity, the GTOS may serve as a rapid screening tool for risk stratification in acute ICU trauma settings when combined with other data.

## 1. Introduction

The intensive care unit (ICU) is a dynamic, high-acuity environment where patients in critical conditions require swift and precise medical intervention. In this setting, healthcare providers must rapidly assess patient severity to make informed decisions regarding treatment and resource allocation. Prognostic scoring systems play a crucial role in this process by providing a standardized method for evaluating patient conditions, enabling clinicians to prioritize interventions, predict outcomes, and ensure appropriate care delivery. The complexity and urgency of ICU care necessitate reliable tools for quick and accurate prognostic assessments. These scoring systems not only aid in the evaluation and stratification of trauma patients but also support a multidisciplinary approach to care. As highlighted by Misseri et al. [1], effective communication and teamwork among surgeons, intensivists, and other healthcare providers are essential for improving patient outcomes in perioperative and critical care settings. By utilizing consistent and reliable scoring systems, healthcare teams establish a common language, facilitate better decision-making, and align all members of the multidisciplinary team in their approach to patient care. This integrated approach improves the quality of care and fosters a more cohesive working environment, ultimately leading to better outcomes for trauma patients in the ICU. Furthermore, by collecting and analyzing patient data, these tools help identify trends and outcomes that may be used to refine treatment protocols and enhance patient care over time. 

Despite their critical role, most ICU scoring systems such as the Acute Physiology and Chronic Health Evaluation (APACHE II) [2], Simplified Acute Physiology Score (SAPS II) [3], and Mortality Prediction Model (MPM II) [4] utilize data obtained on the first day of ICU stay. Research has shown that early predictions based on these scores can sometimes be inaccurate, leading to the development of newer systems that incorporate data from the first three days of ICU hospitalization [5], such as MPM II, the Sepsis-related Organ Failure Assessment (SOFA) [6], the Organ Dysfunction and Infection System (ODIN) [7], Multiple Organ Dysfunction Score (MODS), the Logistic Organ Dysfunction System (LODS) [8], and the Three Days Recalibrated ICU Outcome Score (TRIOS) [9], which provide more dynamic and accurate assessments by reflecting changes in the patient’s condition over time. However, the complexity and extensive data requirements of these systems render them impractical for rapid use in fast-paced ICUs. As a result, there is a growing need for simpler yet reliable prognostic tools that can quickly provide accurate assessments, particularly in ICU trauma cases, where time is essential.

The Geriatric Trauma Outcome Score (GTOS) was first developed to address the unique challenges associated with predicting outcomes in elderly patients with trauma, a population that often presents with multiple comorbidities and reduced physiological reserves [10,11,12,13,14,15]. The GTOS incorporates factors such as age, injury severity, and the need for transfusion within the first 24 h to predict mortality in this vulnerable group. This tool has demonstrated strong predictive power, aiding in treatment decisions, and facilitating discussions between healthcare providers and families regarding appropriate levels of care [13,14]. Although the GTOS was originally designed specifically for elderly patients, its potential application as a rapid and accurate prognostic tool for patients with ICU trauma remains unexplored. This study aimed to evaluate whether the GTOS can serve as an effective tool for risk stratification and mortality prediction across a broader ICU trauma population.

## 2. Methods

### 2.1. Patient Enrollment and Study Design

The procedure was approved by the Chang Gung Memorial Hospital Institutional Review Board (IRB) prior to the start of the study under approval number 202400771B0. The requirement for patient consent was waived due to the retrospective nature of the study. This study reviewed registered medical information from a trauma database [16] in a Level I hospital in southern Taiwan between 1 January 2016 and 31 December 2021. This study included all patients aged ≥20 who were admitted to the ICU due to trauma. The exclusion criteria included burns, hanging, drowning, and incomplete registry data. The research method involved a detailed recording of all retrieved cases’ basic information (age, sex, past medical history, injury body region, and trauma mechanism), blood transfusion history, GTOS, in-hospital mortality, Glasgow Coma Scale (GCS) score, and Injury Severity Score (ISS). The GTOS was calculated by adding the age to the product of ISS multiplied by 2.5, and then adding 22 if any packed red blood cells were transfused within 24 h of arrival.

### 2.2. Statistical Analysis

The chi-square test was performed to determine whether the proportions of categorical variables were the same across the deceased and survivor groups, as well as the odds ratio (OR) and 95% confidence interval (CI) for trauma-related diagnoses, comorbidities, and mortality rates. Homogeneity of variances was first checked using Levene’s test, followed by analysis of variance (ANOVA), to determine the differences between continuous variables within groups. The area under the receiver operating characteristic curve (AUC of ROC) was used to predict mortality performance and determine the optimal cutoff value of the GTOS in determining patient outcomes estimated from the Youden index [17,18], which defines the maximum potential effectiveness of a biomarker. Furthermore, the risk of mortality was calculated after adjusting for potential confounding factors such as age, sex, pre-existing diseases (e.g., hypertension, diabetes, heart disease, end-stage renal disease, and stroke history), and consciousness level using propensity score-matched patient population analysis. Statistical analyses were performed using IBM SPSS Windows version 23. Statistical significance was set at *p* < 0.05.

## 3. Results

### 3.1. Patient Enrollment

**Figure 1:**
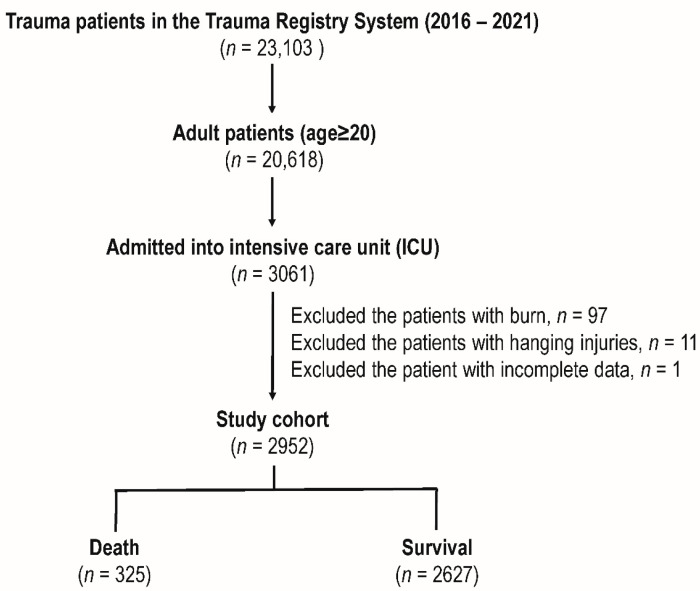
Enrollment of the adult trauma patients administered into the intensive care unit in this study.

The study cohort included 23,103 patients with trauma from the Trauma Registry System (2016–2021). Adult patients (age ≥ 20) were 20,618, of which 3061 were admitted to the ICU. After excluding patients with burns (*n* = 97), hanging injuries (*n* = 11), and incomplete data (*n* = 1), the final study cohort included 2952 patients. Of these, 325 patients died and 2627 survived (Figure 1).

### 3.2. Patient Demographics

Table 1 shows that patients who died had significantly higher odds of being male, older age (63.0 vs. 56.6 years, *p* < 0.001), more blood transfusions (4.5 vs. 1.6 units, *p* < 0.001), higher GTOS (143.2 vs. 110.0, *p* < 0.001), and higher prevalence of comorbidities like hypertension (HTN), coronary artery disease (CAD), and end-stage renal disease (ESRD) compared to survivors. 

The death group had significantly lower median GCS scores (GCS 3–8 group: 66.8 vs. 16.2%, *p* < 0.001), higher rates of severe head/neck and thoracic injuries, higher median ISS (ISS ≥ 25, 75.4 vs. 23.9%, *p* < 0.001), and shorter hospital stays (11.4 vs. 17.3 days, *p* < 0.001). 

### 3.3. The Optimal Cutoff Value of GTOS in Predicting Mortality

**Figure 2:**
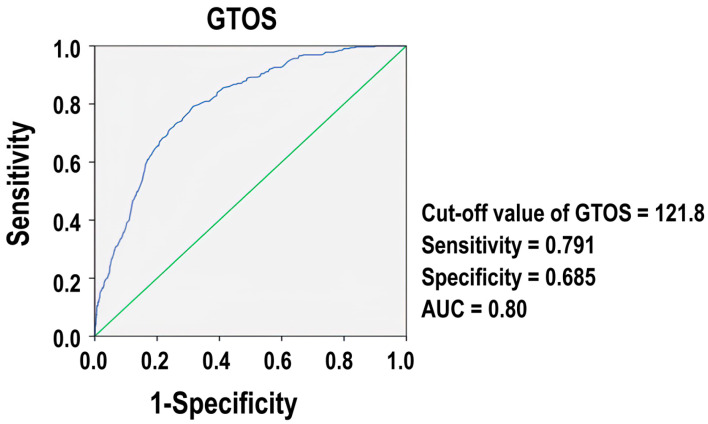
Comparison of the predictive performance of GTOS (blue line). The green line indicates the point where the AUC is equal to 0.50.

Figure 2 presents the ROC for GTOS. The ROC showed that the optimal cutoff value of GTOS was 121.8, at which the sensitivity and specificity were 0.791 and 0.685, respectively. 

The AUC was 0.80, demonstrating that the GTOS is a reasonably accurate measure for assessing mortality in patients with trauma in the ICU, with an AUC value close to 1, indicating excellent predictive power, and a value of 0.5, suggesting no predictive ability.

### 3.4. Comparative Demographics Outcomes Based on Grouping by GTOS

Table 2 compares patients with high (≥121.8) and low (<121.8) GTOS. 

The high GTOS group was significantly older (67.1 vs. 51.6 years, *p* < 0.001), received more blood transfusions (3.4 vs. 1.0 units, *p* < 0.001), and had a higher prevalence of comorbidities, such as HTN, diabetes mellitus (DM), CAD, and ESRD. Patients with a high GTOS had lower median GCS scores (35.2 vs. 14.0%, *p* < 0.001); higher rates of severe head/neck, thoracic, abdominal, and extremity injuries; and a higher median ISS (63.8 vs. 9.6%, *p* < 0.001) than those with a low GTOS. Consequently, mortality was substantially higher in the high-GTOS group (23.7% vs. 3.6%, *p* < 0.001) than in the low-GTOS stay (19.7 vs. 14.9 days, *p* < 0.001).

### 3.5. A Propensity Score-Matched Patient Cohort Analysis

A well-matched propensity score-matched patient cohort is presented in Table 3; there were no significant differences between the groups in terms of sex, age, and several comorbidities. 

Despite adjusting for baseline characteristics, there were still significant differences in the mortality rates and length of hospital stay between the two matched patients. The group with a higher GTOS still experienced higher mortality (17.4% vs. 6.2%, *p* < 0.001) and longer hospital stays (20.3 days vs. 15.3 days, *p* < 0.001).

## 4. Discussion

This study sought to assess the predictive capabilities of the GTOS with a particular focus on its applicability in the ICU environment. Our study found that patients who died were predominantly older, male, required more blood transfusions, scored higher on the GTOS, and had more severe comorbidities compared to survivors. The analysis identified an optimal GTOS threshold of 121.8, yielding an AUC of 0.80 for predicting mortality in ICU trauma patients. Notably, even after adjusting for baseline characteristics, patients scoring 121.8 or higher on the GTOS exhibited significantly higher mortality rates and longer hospital stays than those scoring below this score.

Several studies have validated the predictive accuracy of the GTOS in assessing mortality risk among elderly patients with trauma. Park et al. [19] found that the GTOS was highly effective in the Korean medical context, demonstrating comparable or superior capabilities to the Trauma and Injury Severity Score (TRISS). Meagher et al. [20] reported the predictive performance of the GTOS and GTOS II with the area under the receiver operating characteristic curves (AUROCs) of 0.674 and 0.678, respectively. They also noted the strong predictive power of a GTOS with an AUROC of 0.838, which is superior to conventional scores such as ISS and age [11,13]. Another study by Arslan Erduhan et al. [21] highlights a lower GTOS cutoff point of ≥95 for predicting 30-day mortality in geriatric blunt trauma patients, with a sensitivity of 76% and specificity of 72.27%. In contrast, our study found an optimal GTOS cutoff of 121.8 for predicting overall mortality in a broader ICU trauma population, with higher sensitivity (79.1%) but slightly lower specificity (68.5%). The discrepancy in cutoff values may stem from differences in the study populations, as our cohort included both geriatric and younger ICU trauma patients. Additionally, their focus on 30-day mortality compared to our study’s overall in-hospital mortality likely required a higher GTOS threshold to account for later deaths. Nonetheless, both studies confirmed GTOS as a reliable prognostic tool, with the optimal cutoff varying based on the clinical context, patient population, and outcome measured. In contrast, Ahl et al. [10] showed that the GTOS demonstrated good performance in predicting in-hospital mortality (AUC 0.87), which improved further (AUC 0.88) after excluding patients with treatment limitations or those discharged to hospice care. Additionally, the GTOS showed limited accuracy in predicting the 1-year post-discharge mortality, with a misclassification rate of 17.6%. Thus, the study highlights GTOS’s value of GTOS in acute settings but suggests further adaptations to enhance long-term predictive accuracy.

Although the GTOS can be used as an easy tool for mortality risk stratification in ICU trauma patients, its predictive power for mortality outcomes is not compatible with more complex prognostic scoring tools. Ravindranath et al. [11] compared the GTOS with ICU-specific models such as APACHE III and ANZROD. Their study found the GTOS to be less effective for ICU patients, indicating that, while it is useful for general elderly trauma populations, the GTOS may need refinement in high-acuity ICU settings. Egglestone et al. [22] assessed GTOS effectiveness in an ICU setting, where it showed a moderate predictive capability with an AUROC of 0.68, which was less effective than the Trauma Audit and Research Network’s Probability of Survival, but comparable to the ISS. Furthermore, a meta-analysis revealed that the GTOS achieved a pooled AUROC of 0.80, slightly lower than the TRISS, but better than the ISS, indicating its reliability yet modest performance relative to other scores in predicting mortality in geriatric trauma patients [23]. These studies imply incorporating factors such as vasopressor use, mechanical ventilation, and severe comorbidities could improve the GTOS performance in ICU settings where patients have dynamic clinical conditions. Modifying the GTOS with additional relevant factors may enhance its predictive accuracy. 

This study had several limitations that warrant consideration. First, its single-center focus may limit the generalizability of the findings to diverse demographics and multicenter environments. The retrospective nature of this study and its reliance on registry data introduce potential biases owing to possible incomplete or inaccurate data entries. Another significant limitation is the lack of direct comparisons with other ICU-specific prognostic models, which restricts our understanding of GTOS’s relative performance of GTOS. In addition, the absence of detailed data on vasopressor use and mechanical ventilation, including specific dosages and durations, limits the ability to fully evaluate the impact of these interventions on patient outcomes. To address these limitations, future research should adopt a multicenter prospective approach to validate GTOS’s effectiveness across various clinical scenarios. Continual evaluation and adaptation of the GTOS are crucial to meet the evolving needs of trauma care. By addressing these limitations and expanding the scope of research, we may develop more comprehensive and accurate prognostic tools for ICU trauma patients, ultimately improving patient outcomes and the quality of care.

## 5. Conclusions

The simplicity of the Geriatric Trauma Outcome Score allows for the quick risk stratification of ICU trauma patients. Although not as accurate as complex models, the GTOS provides a rapid screening method for identifying high-risk patients requiring urgent intervention. The integration of the GTOS into medical practice may enable real-time mortality risk assessment and help optimize triage decisions.

## Figures and Tables

**Table 1:** Patient demographics.

Variables	Death *n* = 325	Survival *n* = 2627	OR (95%CI)	*p*
Sex				0.001
Male, *n* (%)	239 (73.5)	1677 (63.8)	1.57 (1.22–2.04)	
Female, *n* (%)	86 (26.5)	950 (36.2)	0.64 (0.49–0.82)	
Age, years (SD)	63.0 ± 18.0	56.6 ± 19.2	-	<0.001
PRBC, units (SD)	4.5 ± 8.0	1.6 ± 3.7	-	<0.001
GTOS	143.2 ± 28.9	110.0 ± 28.9	-	<0.001
Co-morbidities				
HTN, *n* (%)	143 (44.0)	910 (34.6)	1.48 (1.17–1.87)	0.001
DM, *n* (%)	75 (23.1)	532 (20.3)	1.18 (0.90–1.56)	0.234
CAD, *n* (%)	52 (16.0)	213 (8.1)	2.16 (1.56–3.00)	<0.001
ESRD, *n* (%)	28 (8.6)	69 (2.6)	3.50 (2.22–5.51)	<0.001
CVA, *n* (%)	14 (4.3)	140 (5.3)	0.80 (0.46–1.40)	0.435
CHF, *n* (%)	4 (1.2)	15 (0.6)	2.17 (0.72–6.58)	0.161
GCS, median (IQR)	5 (3–13)	15 (12–15)	-	<0.001
3–8, *n* (%)	217 (66.8)	426 (16.2)	10.38 (8.06–13.7)	<0.001
9–12, *n* (%)	22 (6.8)	280 (10.7)	0.61 (0.39–0.96)	0.029
13–15, *n* (%)	86 (26.5)	1921 (73.1)	0.13 (0.10–0.17)	<0.001
AIS ≥ 2				
Hand/Neck, *n* (%)	287 (88.3)	1971 (75.0)	2.51 (1.77–3.57)	<0.001
Face, *n* (%)	45 (13.8)	426 (16.2)	0.83 (0.60–1.16)	0.271
Thorax, *n* (%)	107 (32.9)	681 (25.9)	1.40 (1.10–1.80)	0.007
Abdomen, *n* (%)	43 (13.2)	468 (17.8)	0.70 (0.50–0.98)	0.039
Extremity, *n* (%)	113 (34.8)	926 (35.2)	0.98 (0.77–1.25)	0.864
ISS, median (IQR)	25 (25–30)	16 (16–24)	-	<0.001
1–15, *n* (%)	17 (5.2)	652 (24.8)	0.17 (0.10–0.28)	<0.001
16–24, *n* (%)	63 (19.4)	1348 (51.3)	0.23 (0.17–0.30)	<0.001
≥25, *n* (%)	245 (75.4)	627 (23.9)	9.77 (7.47–12.77)	<0.001
Hospital stay, days (SD)	11.4 ± 15.9	17.3 ± 15.2	-	<0.001

AIS, Abbreviated Injury Scale; CAD, coronary artery disease; CHF, congestive heart failure; CI, confidence interval; DM, diabetes mellitus; ESRD, end-stage renal disease; GCS, Glasgow Coma Scale; GTOS, Geriatric Trauma Outcome Score; HTN, hypertension; IQR, interquartile range; ISS, injury severity score; OR, odds ratio; PRBC, packed red blood cells; SD, standard deviation.

**Table 2:** Comparative demographics outcomes based on GTOS.

	GTOS			
	≥121.8 *n* = 1084	<121.8 *n* = 1868	OR (95%CI)	*p*
Sex				0.545
Male, *n* (%)	696 (64.2)	1220 (65.3)	0.95 (0.82–1.11)	
Female, *n* (%)	388 (35.8)	648 (34.7)	1.05 (0.90–1.23)	
Age, years (SD)	67.1 ± 15.9	51.6 ± 18.6	-	<0.001
PRBC, units (SD)	3.4 ± 5.6	1.0 ± 3.1	-	<0.001
Co-morbidities				
HTN, *n* (%)	501 (46.2)	552 (29.6)	2.05 (1.75–2.39)	<0.001
DM, *n* (%)	262 (24.2)	345 (18.5)	1.41 (1.17–1.69)	<0.001
CAD, *n* (%)	146 (13.5)	119 (6.4)	2.29 (1.77–2.95)	<0.001
ESRD, *n* (%)	47 (4.3)	50 (2.7)	1.65 (1.10–2.47)	0.015
CVA, *n* (%)	67 (6.2)	87 (4.7)	1.35 (0.97–1.87)	0.073
CHF, *n* (%)	11 (1.0)	8 (0.4)	2.38 (0.96–5.94)	0.055
GCS, median (IQR)	13 (6–15)	15 (13–15)	-	<0.001
3–8, *n* (%)	382 (35.2)	261 (14.0)	3.35 (2.80–4.01)	<0.001
9–12, *n* (%)	117 (10.8)	185 (9.9)	1.10 (0.86–1.41)	0.442
13–15, *n* (%)	585 (54.0)	1422 (76.1)	0.37 (0.31–0.43)	<0.001
AIS ≥ 2				
Hand/Neck, *n* (%)	880 (81.2)	1378 (73.8)	1.53 (1.28–1.84)	<0.001
Face, *n* (%)	171 (15.8)	300 (16.1)	0.98 (0.80–1.20)	0.838
Thorax, *n* (%)	436 (40.2)	352 (18.8)	2.90 (2.45–3.43)	<0.001
Abdomen, *n* (%)	209 (19.3)	302 (16.2)	1.24 (1.02–1.51)	<0.031
Extremity, *n* (%)	478 (44.1)	561 (30.0)	1.84 (1.57–2.15)	<0.001
ISS, median (IQR)	25 (20–29)	16 (13–20)	-	<0.001
1–15, *n* (%)	32 (3.0)	637 (34.1)	0.06 (0.04–0.09)	<0.001
16–24, *n* (%)	360 (33.2)	1051 (56.3)	0.39 (0.33–0.45)	<0.001
≥25, *n* (%)	692 (63.8)	180 (9.6)	16.56 (13.59–20.17)	<0.001
Mortality, *n* (%)	257 (23.7)	68 (3.6)	8.23 (6.22–10.88)	<0.001
Hospital stay, days (SD)	19.7 ± 17.2	14.9 ± 13.9	-	<0.001

AIS, Abbreviated Injury Scale; CAD, coronary artery disease; CHF, congestive heart failure; CI, confidence interval; DM, diabetes mellitus; ESRD, end-stage renal disease; GCS, Glasgow Coma Scale; GTOS, Geriatric Trauma Outcome Score; HTN, hypertension; IQR, interquartile range; ISS, injury severity score; OR, odds ratio.

**Table 3:** A propensity score-matched patient cohort analysis based on the GTOS.

Propensity Score-Matched Patient Cohort								
	GTOS							
	≥121.8 *n* = 706		<121.8 *n* = 706		OR (95%CI)		*p*	Standardized Difference
Male, *n* (%)	454	(64.3)	454	(64.3)	1.00	(0.80–1.24)	1.000	0.00%
Age, years (SD)	62.1	±16.1	62.1	±15.8	-		0.976	−0.16%
CVA, *n* (%)	31	(4.4)	31	(4.1)	1.00	(0.60–1.66)	1.000	0.00%
HTN, *n* (%)	282	(39.9)	282	(39.9)	1.00	(0.81–1.24)	1.000	0.00%
CAD, *n* (%)	57	(8.1)	57	(8.1)	1.00	(0.68–1.47)	1.000	0.00%
CHF, *n* (%)	0	(0.0)	0	(0.0)	-		-	-
DM, *n* (%)	157	(22.2)	157	(22.2)	1.00	(0.78–1.29)	1.000	0.00%
ESRD, *n* (%)	8	(1.1)	8	(1.1)	1.00	(0.37–2.68)	1.000	0.00%
GCS, median (IQR)	15	(9–15)	15	(9–15)	-		0.749	−1.70%
Mortality, *n* (%)	123	(17.4)	44	(6.2)	3.17	(2.21–4.56)	<0.001	-
Hospital stay, days (SD)	20.3	±16.3	15.3	±15.2	-		<0.001	-

CAD, coronary artery disease; CHF, congestive heart failure; CI, confidence interval; DM, diabetes mellitus; ESRD, end-stage renal disease; GCS, Glasgow Coma Scale; GTOS, Geriatric Trauma Outcome Score; HTN, hypertension; IQR, interquartile range; OR, odds ratio.

## Data Availability

The de-identified data could be provided for academic research purposes via the corresponding author.
